# The associations between serum high-density lipoprotein cholesterol levels and malignant behavior in pancreatic neuroendocrine neoplasms

**DOI:** 10.1186/s12944-022-01669-z

**Published:** 2022-07-16

**Authors:** Li Quan, Yongkang Liu, Wenjing Cui, Xinru Wang, Weixiao Zhang, Zhongqiu Wang, Chuangen Guo, Chao Lu, Feixiang Hu, Xiao Chen

**Affiliations:** 1 Department of Laboratory Medicine, Affiliated Hospital of Nanjing University of Chinese Medicine, Nanjing, 210029 China; 2 Department of Radiology, Affiliated Hospital of Nanjing University of Chinese Medicine, Nanjing, 210029 China; 3 Department of Radiology, Nanjing Sir Run Run Hospital, Nanjing Medical University, 210029 Nanjing, China; 4 Department of Radiology, The First Affiliated Hospital of Zhejiang University School of Medicine, Hangzhou, 310006 China; 5 Department of Radiology, Fudan University Shanghai Cancer Center, Shanghai, 200032 China; 6 Department of Oncology, Shanghai Medical College, Fudan University, Shanghai, 200032 China; 7 Institute of Radiation Medicine, Fudan University, Shanghai, 200032 China

**Keywords:** Pancreatic neuroendocrine neoplasms, Cholesterol, High-density lipoprotein, Grade, Lymph metastasis

## Abstract

**Background:**

The role of serum high-density lipoprotein cholesterol (HDL-c) in tumorigenesis are observed in several endocrine-related cancers. However, its role in pancreatic neuroendocrine neoplasms (PNENs) has not been understood. In the current study, the relationship between HDL-c levels and malignant behavior in PNENs was explored.

**Methods:**

One hundred ninety-seven patients with histopathology confirmed PNENs were included. PNENs were divided into three grades (G1, G2 and G3) as 2017 WHO classification based on ki67 index and mitosis count. The demographic data, clinical information, tumor morphological and pathological features (organs invasion, lymph node metastasis, vascular invasion and perineural invasion), and serum tumor biomarkers were collected. The relationships between HDL-c levels and malignant behaviors in PNENs were analyzed using logistic regression analysis. Models were also developed for the identification of high grade PNENs.

**Results:**

The levels of serum HDL-c in G2/G3 tumor were significantly lower than that in G1 tumor (*P* = 0.031). However, no such difference was found between G3 and G1/G2. The proportions of low HDL-c (≤ 0.9 mmol/L) were higher in high-grade PNENs (G2/G3 or G3) than those in low-grade (G1 or G1/G2) (29.0 vs 15.2%, *P* = 0.032; 37.0 vs 20.5%, *P* = 0.023). The risk of G2/G3 tumors in patients with high serum HDL-c levels was decreased (odds ratio (OR) = 0.35, 95% confidence interval (CI): 0.12–0.99). Similarly, the risk of G3 PNENs increased in patients with low HDL-c levels (OR = 2.51, 95%CI:1.12–5.60). HDL-c level was also associated with a high ki67 index (> 55%) (OR = 0.10, 95%CI: 0.02–0.51) and neuroendocrine carcinoma G3 (OR = 0.21, 95%CI: 0.06–0.80). The area under the curve (AUC) of HDL-c + tumor size + age was 0.85 (95% CI: 0.79–0.91) in identifying G2/G3 PNENs, and HDL-c (> 0.9 mmol/L) + tumor size + age had an AUC of 0.77 (95% CI: 0.70–0.84) in identifying G3 PNENs. HDL-c level was associated with lymph node metastasis (OR = 0.24, 95%CI:0.08–0.99).

**Conclusion:**

Serum HDL-c levels were significantly associated with malignant behaviors in PNENs, in particular to tumor grade and lymph node metastasis.

## Introduction

Pancreatic neuroendocrine neoplasms (PNENs), known as the common pancreatic epithelial neoplasms, often cause bad outcome because of the the aggressive behavior [1]. The survival rate of PNEN patients with incomplete resections or with unresectable liver metastases was 15–75% [2]. Surgical resection is one of the curative treatments in localized or oligo-metastatic lesions [3]. The incidence of PNENs is 0.8/10,0000 per year which has raised over the last decades [4]. PNENs were classified into three grades (low, intermediate, and high) according to the 2017 WHO classification [5]. PNEN grade is associated with the choices of treatment strategies [6]. Therefore, assessment of tumor grade and prediction of tumor aggressiveness before intervention have attracted great attention over the last decade. However, the assessment or prediction remains challenging.

Several factors that are correlated with PNENs grade and clinical prognosis have been identified, such as clinical factors, serum biomarkers, and radiological features [7–9]. Old age is related to a decreased overall survival and disease specific survival [10, 11]. Conventional imaging features of PNENs, such as tumor size, margin, enhancement pattern, as well as tumor radiomic characteristics have been used to predict PNEN grades and tumor aggressiveness [9, 12–14]. Serum biomarkers, such as chromogranin A (CgA), neuron-specific enolase (NSE), insulin, circulating tumor cells (CTCs), microRNAs (miRNAs), and cytokines also showed high diagnostic/prognostic utility in PNENs [8]. Briefly, serum CgA and NSE have been used as biomarkers of diagnosis and prognosis; Insulin is a specific biomarker for insulinoma; CTCs are related to PNEN grade and survival rate; serum miR-1290 level has a good performance in identifying pancreatic neuroendocrine carcinoma (PNEC).

The role of cholesterol in cancer risk or development has been reported in epidemiological studies and preclinical researches [15]. High-density lipoprotein cholesterol (HDL-c), a good cholesterol [16], exhibits a non-linear association with cancer occurrence [17]. Actually, the associations between HDL-c levels and tumorigenesis or cancer development have been reported in endocrine-related cancers, such as prostate cancer, epithelial thyroid cancer, ovarian cancer, pancreatic cancer, adrenal and testicular cancer [18]. Similar association is also observed in other malignant tumors, such as gastric cancer, hepatocellular carcinoma, and lung adenocarcinoma [19]. A recent study reported that HDL-c levels are associated with malignant intraductal papillary mucinous neoplasms (IPMNs) [20]. However, the potential relationship between serum HDL-c levels and PNENs is not been clarified. This research aimed to investigate the relationship between serum HDL-c levels and the behaviors in PNENs, especially for tumor grade.

## Materials and methods

### Patients

This retrospective cohort study was approved by the Ethics Committee of the Affiliated Hospital of Nanjing University of Chinese Medicine (2017NL-137-05). Informed consent was waived because of the retrospective design. Two hundred fourteen patients with pathology-proved sporadic PNENs were found between June 2012 and July 2021 in our data house. Those patients only adopted biopsy were not included for analyses (*n* = 17, and 13 of them had liver metastasis). Finally, a total of 197 patients that did not receive any treatment before operation were included for analysis in the current study. The demographics, clinical information, pathological features, and biochemical results were collected from medical records. Tumor morphological features (location, size, width), tumor pathological characteristics (tumor grade, lymphatic metastasis, perineural invasion, vascular and adjacent organ invasion), triglyceride (TG), total cholesterol (TC), HDL-c, low density lipoprotein cholesterol (LDL-c), and fasting plasma-glucose level were obtained. Diabetes mellitus (DM) was determined based on the fasting plasma-glucose level and prior history of DM. Blood biochemical biomarkers were tested within 7 days before the operation. Low HDL-c level was defined if serum HDL-c was lower than 0.9 mmol/L (the first quartile of HDL-c levels in all patients).

### Definition of PNENs grade

PNENs were divided into three grades based on the Ki67 index and mitosis count [5]. Briefly, Grade 1 (G1): Ki-67 ≤ 2 and/or mitosis count < 2/10 high power field (HPF); Grade 2 (G2): Ki-67 index is 3–20 and/or mitosis count is 2–20/10 HPF; Grade 3 (G3): Ki-67 index > 20% and/or mitosis count > 20 per 10 HPF. Then, all PNENs were divided into two groups for statistical analyses: G1 and G2/G3 or G1/G2 and G3. For G3 tumors with clear description of tumor differentiation or morphology (*n* = 34), they were divided into well-differentiated one (NET G3) and poor-differentiated one (NEC G3). Moreover, Ki67 index greater than 55% was used as a threshold for advanced pancreatic endocrine carcinoma because the response rate to first-line chemotherapy was lower or the biological behaviour was unfavorable when ki67 was higher than 55% [21, 22].

### Statistical analysis

The Independent-Samples t test or Mann-Whitney U-test was adopted for continuous data analysis. Chi-squared test or Fisher’s exact test was applied for categorical data. Spearman correlation analysis was used to show the association between HDL-c level and ki67 index. Univariate and multivariate logistic regression analyses were utilized to evaluate the association between HDL-c levels and tumor grade in PNENs. Then the relationships between HDL-c and lymph node metastasis, organs, vascular or neural invasion were analyzed using two-tailed t test or logistic regression analysis. The ability of HDL-c levels in the identification of high grade PNENs was determined by receiver operating characteristic (ROC) curves. *P* <  0.05 was defined as statistical significance.

## Results

### The characteristics of PNENs patients

The characteristics of PNENs patients are summarized in Table 1. Patients with G3 or G2/G3 PNENs were older than those with low-grade PNENs (*p* <  0.05). The tumor size of PNENs with high-grade (G2/G3 or G3) were obviously larger than those in lower-grade group (G1 or G2/G1) (*p* <  0.05). Similar trend was observed for glucose levels (*p* <  0.05). Proportion of low HDL-c level in high-grade PNENs (G2/G3 or G3) was higher than that with low-grade (*p* <  0.05). In addition, the levels of HDL-c in patients with G2/G3 PNENs were significantly lower than those with G1 PNENs (*p* <  0.05). Risk of lymphatic metastasis, vascular or organ invasion, and perineural invasion in high-grade PNENs were higher than those with low-grade (*p* <  0.05).

**Table 1:** Characteristics of PNEN patients

Characteristics	Classification 1			Classification 2		
	G1 (*n* = 66)	G2/G3 (*n* = 131)	*P*-value	G1/G2 (*n* = 151)	G3 (*n* = 46)	*P*-value
Sex (n)			0.245			0.003
Male	31	73		71	33	
Female	35	58		80	13	
Age (yr)	53.50 ± 11.38	57.34 ± 11.62	0.029	54.73 ± 11.81	60..41 ± 10.06	0.002
Location			0.175			0.668
Head-neck	37	56		69	24	
Body	17	49		53	13	
Tail	12	26		29	9	
Tumor size (cm)^a^	1.5(1.2–2.43)	3.5(2.5–4.9)	< 0.001	2.5(1.5–3.9)	3.65(2.98–5.5)	< 0.001
Lymph			0.026			0.004
Yes	2	17		9	10	
No	64	114		142	36	
Vascular invasion			0.001			< 0.001
Yes	2	26		14	14	
No	64	105		137	32	
Organs invasion			< 0.001			0.044
Yes	0	29		18	11	
No	66	102		133	35	
Neural invasion			0.019			0.009
Yes	2	18		10	10	
No	64	113		141	36	
Glu(mmol/L)	5.12 ± 1.09	5.75 ± 2.09	0.009	5.49 ± 1.98	5.69 ± 1.24	0.036
TG (mmol/L)	1.41 ± 0.87	1.37 ± 0.83	0.494	1.37 ± 0.86	1.42 ± 0.80	0.712
TC (mmol/L)	4.49 ± 1.00	4.27 ± 1.09	0.173	4.30 ± 1.03	4.49 ± 1.14	0.206
HDL-c (mmol/L)	1.23 ± 0.37	1.11 ± 0.36	0.031	1.16 ± 0.35	1.09 ± 0.41	0.511
HDL-c ≤ 0.9 (mmol/L)	10	38	0.032	31	17	0.023
HDL-c > 0.9 (mmol/L)	56	93		120	29	
LDL (mmol/L)	2.56 ± 0.75	2.39 ± 0.84	0.183	2.45 ± 0.80	2.42 ± 0.87	0.928
DM						0.367
Yes	10	32		30	12	
No	56	99		121	34	

*DM* Diabetes mellitus, *Glu* Glucose, *HDL-c* High density lipoprotein-cholesterol, *PNEN* Pancreatic neuroendocrine neoplasm, *TG* Triglyceride, *TC* Total cholesterol

^a^Data was shown as median (IQR) and analyzed using Mann-Whitney U-test

**Fig. 1:**
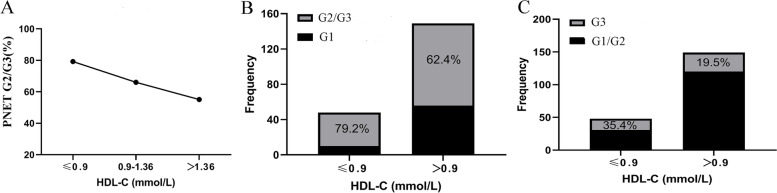
The association between high density lipoprotein-cholesterol (HDL-c level) and pancreatic neuroendocrine neoplasm (PNEN) grades. **A** The proportion of G2/G3 PNENs decreased with the increase of HDL-c level; **B**, **C** The proportion of high-grade PNENs (G2/G3 (**B**); G3 (**C**)) in low HDL-c was more common than those with high HDL-c level

The prevalence of G2/G3 PNENs were reduced with the elevation of HDL-c levels (*p* <  0.05) (Fig. 1A). The proportion of G2/G3 PNENs in patients with low HDL-c level was higher than those with high HDL-c level (79.2 vs 62.4%) (Fig. 1B). Similarly, the proportion of G3 PNENs in patients with low HDL-c level was higher than those with high HDL-c level (35.4 vs 19.5%) (Fig. 1C).

### HDL-c levels and PNEN grades

HDL-c levels were negatively correlated to ki67 index (*r* = − 0.14, *p* = 0.045). Moreover, the associations between HDL-c levels and PNEN grades were evaluated using logistic analysis (Table 2). HDL-c level was associated with G2/G3 tumors (univariate, odds ratio (OR) = 0.41, 95% confidence interval (CI): 0.18–0.93; multivariate, OR = 0.35, 95%CI:0.12–1.00). Moreover, low HDL-c (< 0.9 mmol/L) level was also associated with G3 tumor (univariate, OR = 2.27, 95% CI: 1.11–4.65; multivariate, OR = 2.51, 95%CI:1.12–5.60). Age, tumor size and vascular invasion were also independent associated factors for high-grade PNENs (*p* < 0.05 or 0.01). In addition, HDL-c level was also associated with a high ki67 index (> 55.0%) (univariate, OR = 0.19, 95% CI: 0.05–0.80; multivariate, OR = 0.10, 95%CI:0.02–0.51) and NEC G3 (*n* = 28) (univariate, OR = 0.27, 95% CI: 0.08–0.93; multivariate, OR = 0.21, 95%CI:0.06–0.80) (Table 3).

**Table 2:** Associated factors of PNENs grade

Variables	G2/G3 vs G1 (model 1)				G3 vs G1/G2 (model 2)			
	Univariate OR (95%CI)	*p*	Multivariate OR (95%CI)	*p*	Univariate OR (95%CI)	*p*	Multivariate OR (95%CI)	*p*
Age (year)	1.03 (1.00–1.06)	0.031	1.05 (1.02–1.09)	< 0.01	1.05 (1.02–1.08)	< 0.01	1.06 (1.02–1.10)	< 0.01
Tumor size (cm)	2.51 (1.83–3.43)	< 0.01	2.46 (1.76–3.46)	< 0.01	1.31 (1.12–1.53)	< 0.01	1.30 (1.10–1.55)	< 0.01
HDL-c (mmol/L)	0.41 (0.18–0.93)	0.033	0.35 (0.12–1.00)	0.049	0.56 (0.22–1.41)	0.22	/	
HDL-c (≤ 0.9 vs > 0.9 mmol/L)	2.29 (1.06–4.95)	0.035	/		2.27 (1.11–4.65)	0.025	2.51 (1.12–5.60)	0.02
Glucose level (mmol/L)	1.38 (1.04–1.82)	0.025	1.18 (0.85–1.64)	0.35	1.06 (0.89–1.25)	0.52	1.01 (0.84–1.21)	0.91
Vascular invasion	7.92 (1.82–34.51)		5.06 (1.02–25.23)	0.048	4.28 (1.86–9.87)	< 0.01	3.85 (1.53–9.70)	< 0.01

“/” means that the variables were not included in the regression analysis

*CI* Confidence interval, *HDL-c* High density lipoprotein-cholesterol, *PNENs* Pancreatic neuroendocrine neoplasms

**Table 3:** Associated factors of advanced PNENs (Ki67 index > 55% or NEC G3)

Variables	Ki67 index > 55%				NEC G3^a^			
	Univariate OR (95%CI)	*p*	Multivariate OR (95%CI)	*p*	Univariate OR (95%CI)	*p*	Multivariate OR (95%CI)	*p*
Age (year)	1.07 (1.02–1.12)	0.031	1.09 (1.03–1.14)	< 0.01	1.05 (1.02–1.09)	0.01	1.06 (1.02–1.11)	< 0.01
Tumor size (cm)	1.25 (1.03–1.53)	0.33	1.36 (1.06–1.74)	0.21	1.30 (1.10–1.53)	0.33	1.36 (1.12–1.65)	< 0.01
HDL-c (mmol/L)	0.19 (0.05–0.80)	0.023	0.10 (0.02–0.51)	0.01	0.27 (0.08–0.93)	0.04	0.21 (0.06–0.80)	0.02
Glucose level (mmol/L)	1.07 (0.88–1.32)	0.49	0.99 (0.77–1.27)	0.99	1.06 (0.88–1.28)	0.56	0.98 (0.79–1.22)	0.84
Vascular invasion	3.96 (1.41–11.09)	0.02	3.58 (1.14–11.21)	0.040	2.31 (0.87–6.14)	0.09	1.82 (0.63–5.26)	0.27

*CI* Confidence interval, *PNENs* Pancreatic neuroendocrine neoplasms, *NEC* Neuroendocrine carcinoma

^a^*n* = 185 (G1 = 66, G2 = 85, NET G3 = 6, NEC G3 = 28)

### ROC analysis

**Fig. 2:**
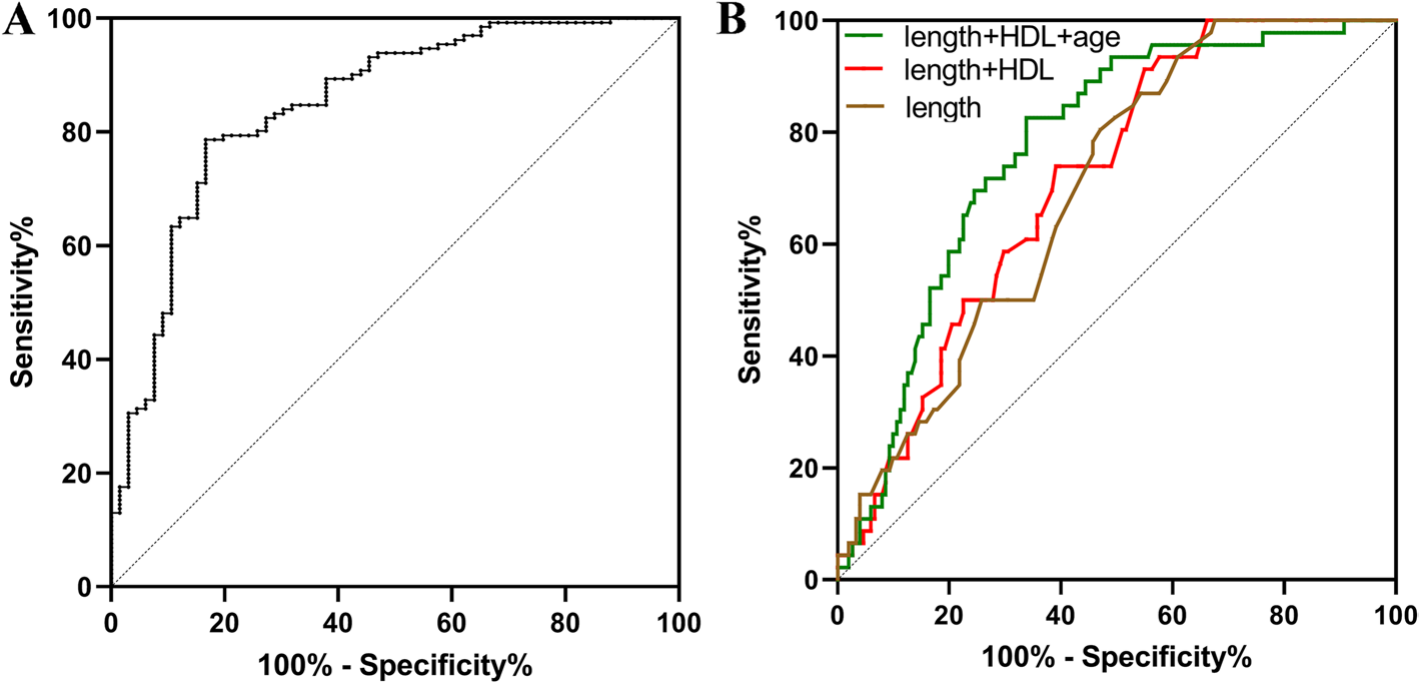
Receiver operating characteristic analysis to differentiate the high grade pancreatic neuroendocrine neoplasms (PNENs) from the low grade PNENs. **A** Performance of high density lipoprotein-cholesterol (HDL-c) combined with age and tumor size in recognizing G1 tumor from G2/G3 tumor; **B** Performance of tumor size alone, HDL-c combined with tumor size, HDL-c combined with age and tumor size in recognizing G1/G2 tumor from G3 tumor

The performance of variables in identifying high-grade PNENs (G2/G3 or G3) is shown in Fig. 2. The area under the curve (AUC) of HDL-c plus tumor size and age was 0.85 (95% CI: 0.79–0.91) in identifying G2/G3 PNENs (Fig. 2A). The AUCs of tumor size, high HDL-c level (> 0.9 mmol/L) plus tumor size, high HDL-c (> 0.9 mmol/L) plus tumor size and age were 0.70 (95% CI: 0.62–0.77), 0.71 (95% CI: 0.63–0.78), 0.77 (95% CI: 0.70–0.84) in identifying G3 PNENs (Fig. 2B), respectively.

### HDL-c levels and PNENs metastasis risk

**Fig. 3:**
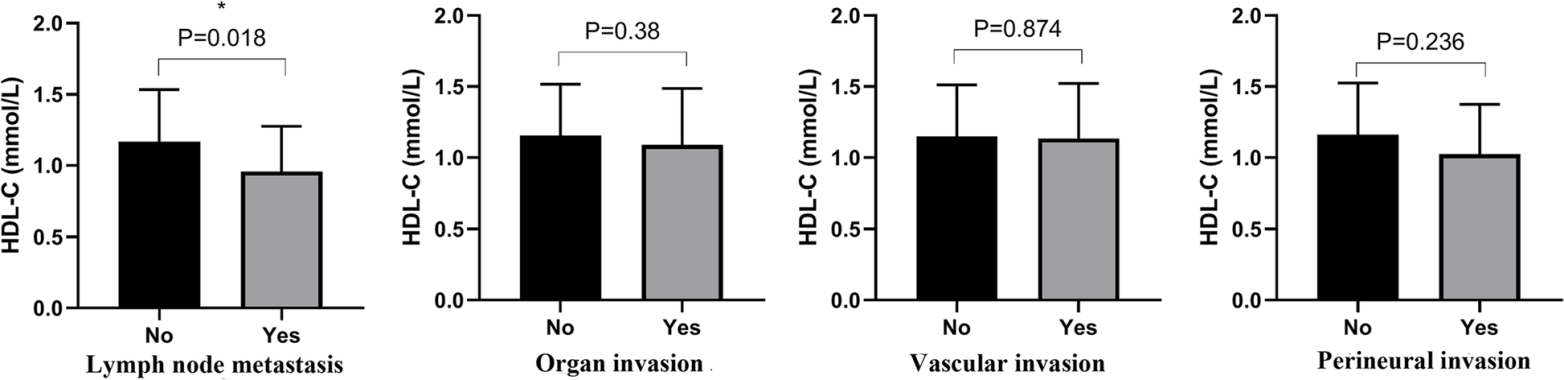
The high density lipoprotein-cholesterol (HDL-c) levels in pancreatic neuroendocrine neoplasms patients with and without lymph invasion, organs invasion, vascular invasion and neural invasion

The HDL-c level in patients with lymph node invasion was significantly lower than that without lymph node invasion (*p* = 0.018) (Fig. 3), but no such trends were observed in organs invasion, vascular and perineural invasion (Fig. 3). Moreover, HDL-c level was an independent associated factor for lymph node metastasis after adjusting with tumor grade and tumor size (OR = 0.24, 95%CI: 0.58–0.99; OR = 0.21, 95%CI:0.05–0.91; OR = 0.23, 95%CI:0.06–0.94) (Table 4).

**Table 4:** Associated factors of lymph node metastasis

Variables	Model 1				Model 2				Model 3			
	Univariate OR (95%CI)	*p*	Multivariate OR (95%CI)	*p*	Univariate OR (95%CI)	*p*	Multivariate OR (95%CI)	*p*	Univariate OR (95%CI)	*p*	Multivariate OR (95%CI)	*p*
HDL-c (mmol/L)	0.18 (0.05–0.76)	0.02	0.24 (0.08–0.99)	0.048	0.18 (0.05–0.76)	0.019	0.21 (0.05–0.91)	0.037	0.18 (0.05–0.76)	0.019	0.23 (0.06–0.94)	0.04
Tumor size (cm)	1.24 (1.05–1.47)	0.01	1.16 (0.96–1.42)	0.13	1.24 (1.05–1.47)	0.13	1.19 (0.98–1.44)	0.073	1.24 (1.05–1.47)	0.013	1.19 (0.98–1.45)	0.084
Grade 1	0.11 (0.02–0.54)	< 0.01	0.20 (0.04–1.05)	0.057	/		/		/		/	
Grade 2	0.32 (0.11–0.92)	0.03	0.38 (0.13–1.11)	0.076	/		/		/		/	
Grade 3	1 (reference)		1 (reference)		/		/		/		/	
G1	/		/		0.21 (0.05–0.94)	0.04	0.36 (0.07–1.72)	0.198	/		/	
G2 + G3	/		/		1 (reference)		1 (reference)		/		/	
G1 + G2	/		/		/		/		0.23 (0.09–0.60)	< 0.01	0.32 (0.11–0.90)	0.03
G3	/		/		/		/		1 (reference)		1 (reference)	

Model 1: grade were divided into three groups (G1, G2 vs G3 (reference)); Model 2: grade were divided into two groups (G1 vs G2/G3 (reference)); Model 3: grade were divided into two groups (G1/G2 vs G3 (reference))

“/” means that the variables were not included in the regression analysis

*CI* Confidence interval

## Discussion

Increasing evidence suggests that the cholesterol plays noticeable role in tumorigenesis and cancer progression [23]. Lipoproteins are also markers for monitoring cancer progression [19]. As one of the major components, HDL-c showed a meaningful correlation with cancer risk [24–32]. To our knowledge, few studies have observed the associations between HDL-c level and PNENs, especially for the tumor grade and metastasis. In this study, the occurrence of low HDL-c level was associated with PNENs grade. Multivariate logistic regression analyses also displayed that low HDL-c levels were related to high-grade PNENs. Combined HDL-c level and other clinical characteristics showed high performance to identify the high grade PNENs. Additionally, this study reported that HDL-c level was an independent associated factor of lymph node metastasis.

Histopathological grade is an important factor for treatment strategies in PNENs. Surgical or conservative approach should be performed for different grade of PNENs [3]. In addition, histological grade is associated with overall prognosis as well as post-surgical recurrence and mortality [7]. Several studies showed that quantitative radiographic features [9, 12] and serum biomarkers [8] could be used for PNENs grading. However, few studies have observed the reparations between HDL-c level and PNEN grade. A recent study investigated the association between lipids levels and risk of PNENs [33]. Univariate analysis showed that hypertriglyceridemia (OR = 2.43, 95%CI: 1.28–4.60), not hypercholesterolemia and low levels of HDL cholesterol (OR = 1.91, 95%CI:0.87–4.22), was associated factor for PNENs occurrence. The data of the present study showed that HDL-c levels were independently associated with high-grade PNENs. Interestingly, a recent study also reported that low HDL-c level was associated with malignancy in IPMNs [20]. Wu et al. reported that serum apolipoprotein A1, one major HDL-c constituent, was correlated with larger tumor size, tumor differentiation, and poor histological grade [34]. How HDL-c affects the PNENs is not totally clarified. HDL-related apolipoproteins and enzymes may play important roles in antioxidant, anti-angiogenesis, anti-inflammatory, anti-apoptosis and anti-tumorigenesis [35]. Tumor microenvironment (TME) is also related to tumor progression [17], and HDL-c can affect cell components of the TME through several signal pathways [36]. In addition, the demand for cholesterol in tumors with high proliferative ability is high, which promotes lipid internalization and lipoprotein consumption, and consequently causes a decrease of HDL-c level [37]. High-grade PNENs usually had high proliferative activity which may need more cholesterol for membrane synthesis. Moreover, high-grade PNENs with large tumor size may affect the exocrine function of pancreas or secret hormones, and affects the nutritious status [38] or metabolism [39], which may influence the HDL-c levels. Malnutrition is a common comorbidity in patients with PNENs [38]. Low HDL-c level is one indicator of metabolic syndrome (MtS). Interestingly, MtS is also related to poor clinical outcome of PNENs [39], which partly supported the findings in the present study.

Lymph node metastasis is associated with clinical outcome for patients with PNENs [40]. However, it is still difficult to determine whether the patient has lymph node metastasis before operation [8]. Findings in this study hinted that serum HDL-c levels were independently associated with lymphatic metastasis after adjusting for tumor size and histopathological grade, which suggested that HDL-c could be considered as a preoperative candidate for predicting lymphatic metastasis. Actually, HDL-c links to lymph node metastasis has been reported in other type of cancers. Lymphatic invasion was observed more often in gastric cancer patients with low HDL-c level [41]. Moreover, serum HDL-c levels was well associated with nodal stages [42]. HDL-c may inhibit caner metastasis from lymph node by anti-tumorigenesis. However, HDL-c levels were not associated with vascular or organs invasions. The reason for this phenomenon is unclear. Further population studies and experimental researches are needed to explore the possible mechanisms. .

### Comparisons with other studies and what does the current work add to the existing knowledge

HDL-c levels may be related to cancer occurrence or progression. However, the role of serum HDL-c levels in PNENs grade or malignant behavior is still unknown. The current study firstly reported that low HDL-c level was a biomarker for high grade PNENs and lymph node metastasis. The results of this study may be useful for PNENs management.

### Study strengths and limitations

This study has several limitations. First, some factors may be associated with serum lipid levels, such as smoking or weight. However, such associated factors were not controlled in our mutivariate analysis. Second, this study only observed the association, but the possible mechanisms are not studied and are unclear. Third, we developed models to predict high-grade PNENs. However, addition of HDL-c only slightly improved the performance of tumor size and age. Fourth, the recent new WHO grading classification (2019 or 2022) for PNENs were not used in this study. Finally, we did not observe the relationships between serum HDL-c levels and PNEN prognosis.

## Conclusions

This study showed an independent association between serum HDL-c levels and malignant PNENs, especially for tumor grade and lymph node metastasis. The models based on HDL-c, tumor size and age had good performance in identifying high-grade PNENs. HDL-c level may be an useful factor for PNENs clinical management.

## Data Availability

All data generated or analyzed during this study are included in this published article (and its Supplementary Information files).

## Abbreviations

AUC: Area under the curve
CI: Confidence interval
DM: Diabetes mellitus
HDL-c: High density lipoprotein-cholesterol
LDL: Low density lipoprotein
OR: Odds ratio
PNENs: Pancreatic neuroendocrine neoplasms
ROC: Receiver operating characteristic
TG: Triglyceride
TC: Total cholesterol
